# Appropriate Age for Height Control Treatment in Patients With Marfan Syndrome

**DOI:** 10.3389/fendo.2021.708931

**Published:** 2021-10-21

**Authors:** Sung Eun Kim, Dong-Yun Lee, Min-Sun Kim, Sung Yoon Cho, Dong-Kyu Jin, DooSeok Choi

**Affiliations:** ^1^Department of Obstetrics and Gynecology, Samsung Medical Center, Sungkyunkwan University School of Medicine, Seoul, South Korea; ^2^Department of Pediatrics, Samsung Medical Center, Sungkyunkwan University School of Medicine, Seoul, South Korea

**Keywords:** Marfan syndrome, height control, estrogen treatment, estradiol valerate, cut-off age

## Abstract

**Objective:**

This study aimed to determine the most appropriate age for height control treatment in patients with Marfan syndrome (MFS).

**Materials and Methods:**

This retrospective study included patients with MFS who underwent height control treatment with estradiol valerate. The estrogen dose was increased according to the height change. The cut-off age for the maximum difference between the expected height and actual final height was evaluated.

**Results:**

Seventeen patients were included in this study. The difference between the height predicted by the growth curve and the final height (gcHtD) and that predicted by the bone age and the final height (baHtD) was the largest in the 10.5 years age group (p=0.0045 and p=0.0237, respectively). The gcHtD was 10.6 (10.2, 13.5) cm for patients aged ≤10.5 years, whereas it was 0.6 (−3.65, 5.85) cm for patients aged >10.5 years. The baHtD was 10.1 (7.31, 11.42) cm for patients aged ≤10.5 years, while it was 3.83 (0.84, 6.4) cm for patients aged >10.5 years. When height change was observed for a minimum of 6 months after completion of estrogen treatment, the average growth was 0.6 (0.2, 2.1) cm.

**Conclusion:**

Initiating height control treatment before the age of 10.5 years is effective in female patients with MFS.

## Introduction

Marfan syndrome (MFS) is a genetic disorder of the connective tissue, mostly occurring due to a mutation in the fibrillin-1 gene (1), and affects 1/5,000 to 1/10,000 individuals (2, 3). MFS is characterized by ectopia lentis, aortic aneurysm, skeletal defects, arachnodactyly, pectus deformity, tall stature, and joint hypermobility (1). Tall stature may lead to social adjustment problems in patients with MFS (4). Therefore, height control treatment is considered for some MFS patients.

Since puberty-associated growth spurt progresses rapidly in patients with MFS, estrogen treatment before the peak height period was considered to be helpful for height control (5). However, some studies have suggested that estrogen treatment does not control the height. Trygstad et al. considered estrogen treatment for height control a waste of time (6). In a study by Rozendaal et al., ethinyl estradiol treatment did not have a statistically significant effect on reducing growth after adjusting for confounding factors (7).

However, in a study evaluating the effect of estrogen treatment according to age, the treatment outcome seemed to be better when the treatment was initiated earlier, especially before puberty (8). In another study, although the statistical significance could not be investigated due to the small sample size, treatment before the age of 11 years was considered effective for height control (9). Therefore, it was considered that the treatment effect varies according to the age at treatment initiation. However, no study has investigated the appropriate age for initiating height control treatment in patients with MFS.

Therefore, this study aimed to determine the most appropriate age for initiating estrogen treatment for height control in patients with MFS.

## Materials and Methods

### Study Design

From January 2000 to December 2020, all patients diagnosed with MFS who were referred to the Department of Obstetrics and Gynecology for height control treatment and had completed treatment were included. At first, the height predicted by the growth curve or bone age was informed to each patient, and after sufficient consultation, treatment was started only who desired shorter than predicted height. We included a total of 17 female patients who had completed treatment, excluding those with missing data on bone age measurements at either treatment initiation or completion (n = 4) and those who were lost to follow-up (n = 6).

This study was approved by the Institutional Review Board of Samsung Medical Center (IRB no. 2021-01-069). Since this was a retrospective study using medical chart data, the requirement for informed consent was waived.

### Height Prediction

Height was predicted by referring to the chronological age and bone age at treatment initiation. The expected height was determined by referring to a method using the Marfan growth curve (10) and bone maturation (11). The height of the patient after treatment completion was measured, and the difference between the height based on the growth curve and the height according to the bone age, which was initially predicted, was calculated. Since the patient may have been 1–2 cm taller after discontinuing the medication, the height was remeasured through follow-up observation for >6 months.

### Treatment Regimen

The drug administration method was applied in the same manner for all patients. First, estradiol valerate (Progynova^®^, Bayer Schering Pharma AG, Germany) 2 mg was administered for 6 months. Thereafter, the dose of estradiol valerate was increased by 2 mg every 2 months and up to a maximum dose of 12 mg/day. If the change in height was <1 cm, the estradiol dose was maintained. If no change in height was observed for 6 months (three consecutive outpatient visits), patients underwent radiographic assessment to evaluate fusion of the epiphyseal plate. When bone maturation was completed, the dose of estradiol valerate was decreased by 2 mg every 2 months. And eventually, patients stopped taking estradiol valerate. To protect endometrium, progesterone withdrawal bleeding was induced. Six months after initiating estradiol valerate treatment at 4 mg or if breakthrough bleeding occurred in the first 6 months of treatment, medroxyprogesterone acetate (Provera^®^, Pfizer, USA) 10 mg/day was administered from day 1 to 12 of each month.

### Statistical Analysis

Characteristics are presented as frequency (percentage) for categorical variables and median (interquartile range [IQR]) for continuous variables. The differences between groups were compared using the Wilcoxon rank-sum test or Fisher’s exact test as appropriate. The cut-off point for detecting the significant maximum difference between final height and expected height by growth curve or bone age was determined and calculated using the Wilcoxon rank-sum test. The differences between the young and older groups stratified according to the cut-off age were plotted using a box plot. Two-sided p-values of <0.05 were considered statistically significant. All analyses were performed using SAS software, version 9.4 (SAS Institute Inc., Cary, NC, USA), and R software, version 3.6.3 (R Project for Statistical Computing).

## Results

The age at treatment initiation ranged from 8 to 12 years and 9 months. The analysis included variables such as menarche, Tanner stage, serum estradiol level, height at treatment initiation, predicted height by the growth curve, predicted height by the bone age, difference between the height predicted by the growth curve and the final height (gcHtD), and difference between the height predicted by the bone age and the final height (baHtD).

Based on the chronological age at treatment initiation, 9 years and 6 months was converted to 9.5 years. By dividing the age data into 0.1 intervals, we found an age at which gcHtD and baHtD were statistically significant. At the age of 10.5 years, the p-value for gcHtD and baHtD was 0.0045 and 0.0237, respectively. At the age of 11 years, the p-value for gcHtD and baHtD was 0.0138 and 0.0307, respectively. Although statistical significance was observed at both ages, there was a maximum difference at the age of 10.5 years. Similarly, we compared whether there was a significant difference in height based on the bone age at treatment initiation. However, a significant difference in height according to the bone age was observed only in patients aged 10–11 years (p = 0.0141).

**Figure 1:**
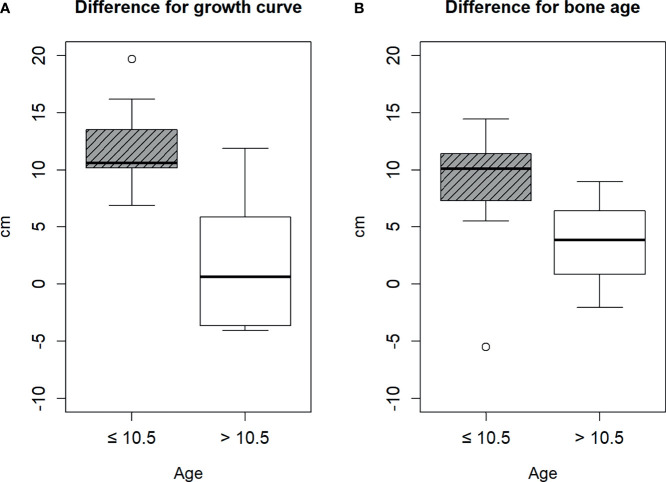
Difference between the estimated height and final height. **(A)** by growth curve, **(B)** by bone age.

Therefore, we divided the patients into two groups based on the cut-off age of 10.5 years. The age of nine and eight patients was ≤10.5 and >10.5 years, respectively. There was no statistical difference between the baseline characteristics of patients ≤10.5 and >10.5 years, except height, bone age, gcHtD, and baHtD (**Table 1**). The gcHtD was 10.6 (10.2, 13.5) cm for patients aged ≤10.5 years, whereas it was 0.6 (−3.65, 5.85) cm for patients aged >10.5 years. The baHtD was 10.1 (7.31, 11.42) cm for patients aged ≤10.5 years, while it was 3.83 (0.84, 6.4) cm for patients aged >10.5 years old (**Figure 1**).

**Table 1:** Comparison of baseline characteristics of two groups divided by age.

	Age ≤ 10.5 (n=9)	Age > 10.5 (n=8)	P-value
Chronological age (year)	8.82 [8.54, 9.74]	11.6 [11.25, 11.9]	*0.0006
Bone age (year)	8.83 [8.83, 10]	11.25 [10.5, 12.5]	0.0294
Menarche			1.0000
Yes	0	1	
No	8	7	
Unknown	1	0	
Tanner stage			0.2559
I	5	4	
II	3	1	
III	0	3	
Serum E2 (pg/mL)	10 [3, 27]	14.5 [2, 51.5]	0.5294
Height at the beginning of treatment (cm)	151.2 [147.7, 155.8]	158.75 [154.75, 169.75]	0.0922
gcHtP (cm)	184 [181, 186]	171.5 [167, 184]	0.0740
baHtP (cm)	183.92 [177.71, 185.75]	173.87 [172.71, 180.19]	0.1629
gcHtD (cm)	10.6 [10.2, 13.5]	0.6 [-3.65, 5.85]	*0.0045
baHtD (cm)	10.1 [7.31, 11.42]	3.83 [0.84, 6.4]	*0.0237
Height at the last medication	171.5[170.4, 173.6]	172.95[170.7, 176.2]	0.5964
Last height	173.1[171.5, 173.4]	174.5[171, 177.8]	0.6650
FHtD (cm)	0.6[0.2, 1.2]	0.5[0.1, 2.2]	1.0000
Treatment period (month)	50 [44, 55]	44 [30, 49]	0.2479

Characteristics were described as median (interquartile range, IQR).

E2; estradiol, gcHtP; predicted height by growth curve, baHtP; predicted height by bone age, gcHtD; height difference between final and predicted height by growth curve, baHtD; height difference between final and predicted height by bone age, FHtD; Final height difference (Last height – Height at the last medication).

*Statistically significant (p < 0.05).

A difference in height may be observed after discontinuation of the medication; therefore, the height was reassessed after 6 months, revealing a height difference of 0.6 (0.2, 2.1) cm. Ten patients showed a height difference of >1 cm, two patients showed a height difference of 1–2 cm (1.1 and 1.2 cm), and five patients showed a height difference of >2 cm (2.1, 2.1, 2.3, 3.6, and 4.2 cm). There were no cases of additional spine-related surgery or treatment after completion of estrogen treatment.

## Discussion

In this study, a cut-off age that showed significant height difference based on chronological age was 10.5 years. Furthermore, initiating treatment before the age of 10.5 years was found to be effective for height control. Regarding bone age, treatment between the age of 10 and 11 years was meaningful in terms of the difference between the final height and the post-treatment height.

Patients with MFS may have a psychological burden and problems with social adjustment because of their height (4). Thus, after predicting the height based on the bone age or growth curve, if a patient is expected to be too tall, height control treatment may be considered.

Estrogen treatment is effective in controlling height. Height control by estrogen treatment was first started in 1956 (12). Since then, several studies have been conducted using various types of estrogen formulations and dosages. However, some studies have shown that estrogen treatment does not control the height. One study including 680 tall girls showed that estrogen treatment after puberty had no effect on height control (6). In a study initiating estrogen treatment around the age of 12 years, the final height after treatment did not decrease significantly compared to the prediction height (13). In another study, the effect of ethinyl estradiol on growth reduction was not statistically significant (7).

However, because patients with MFS have an earlier and more intense peak height growth velocity at puberty than the general population (5), early treatment for height control has been suggested. In the abovementioned studies, there was no effect on growth when the treatment was initiated after the age of 11.4 years (7) or after puberty (6). Although a study included only four patients, it showed that initiating treatment at the age of 10.6 years was effective in controlling height (14). Although statistical significance could not be achieved because the small sample size was small, one study of eight patients with estradiol valerate suggested that treatment initiation before the age of 11 years is effective for height control (9). Moreover, there have been insufficient studies on the appropriate age for effective height control in patients with MFS.

It is difficult to conduct research because of the few number of patients with MFS and not all patients receive hormone therapy for height control. From 1969 to 2002, only 22 patients were treated in four centers in the Netherlands (7). Further, in one center, only four patients were treated over 16 years (14). Therefore, this study is meaningful as it enrolled 17 patients who completed height control treatment from a single center.

This study revealed that there were cases in which the height was increased after treatment completion, although it was judged that bone maturation had halted after treatment. Thus, it is necessary to inform the patient that a change in height of about 1–2 cm can occur even after treatment completion.

Even though there was no case of thromboembolic event in our study, it is necessary to consult enough about the risks at the time of counseling before starting treatment. In addition, in a previous study, there was an effect on infertility in women who used ethinyl estradiol 200 μg (15). However, since the regimen we used is estradiol valerate, it is difficult to say that estradiol valerate has the same effect on fertility. The outcome for the patient’s fertility should be tracked through a long-term follow-up.

The strength of our study is that, for the first time, a statistically significant cut-off age for effective height control was suggested. Unlike other studies, this study evaluated changes in height after treatment completion. However, this study has a limitation; the absolute number of patients included in the study was small. In addition, since it is thought that patients who did not receive treatment would have a shorter expected height, there is also a bias in patient selection. A follow-up prospective multicenter study with a large number of patients is recommended in the future.

In conclusion, for the most effective height control in patients with MFS, initiating estrogen therapy before the chronological age of 10.5 years and between the bone age of 10 and 11 years should be considered.

## Data Availability Statement

The raw data supporting the conclusions of this article will be made available by the authors, without undue reservation.

## Ethics Statement

The studies involving human participants were reviewed and approved by Institutional Review Board of Samsung Medical Center. Written informed consent from the participants’ legal guardian/next of kin was not required to participate in this study in accordance with the national legislation and the institutional requirements.

## Author Contributions

All authors listed have made a substantial, direct, and intellectual contribution to the work and approved it for publication.

## Conflict of Interest

The authors declare that the research was conducted in the absence of any commercial or financial relationships that could be construed as a potential conflict of interest.

## Publisher’s Note

All claims expressed in this article are solely those of the authors and do not necessarily represent those of their affiliated organizations, or those of the publisher, the editors and the reviewers. Any product that may be evaluated in this article, or claim that may be made by its manufacturer, is not guaranteed or endorsed by the publisher.
